# Serum Cytokines Usefulness for Understanding the Pathology in Allergic Bronchopulmonary Aspergillosis and Chronic Pulmonary Aspergillosis

**DOI:** 10.3390/jof8050436

**Published:** 2022-04-23

**Authors:** Yuya Ito, Takahiro Takazono, Yasushi Obase, Susumu Fukahori, Nobuyuki Ashizawa, Tatsuro Hirayama, Masato Tashiro, Kazuko Yamamoto, Yoshifumi Imamura, Naoki Hosogaya, Chizu Fukushima, Yoshitomo Morinaga, Katsunori Yanagihara, Koichi Izumikawa, Hiroshi Mukae

**Affiliations:** 1 Department of Respiratory Medicine, Nagasaki University Hospital, 1-7-1, Sakamoto, Nagasaki 852-8501, Japan; y.ito@nagasaki-u.ac.jp (Y.I.); takahiro-takazono@nagasaki-u.ac.jp (T.T.); susumu-f@nagasaki-u.ac.jp (S.F.); nashizawa-ngs@umin.ac.jp (N.A.); tatsuro_h_20@nagasaki-u.ac.jp (T.H.); mtashiro@nagasaki-u.ac.jp (M.T.); kazukomd@nagasaki-u.ac.jp (K.Y.); f-chizu@nagasaki-u.ac.jp (C.F.); hmukae@nagasaki-u.ac.jp (H.M.); 2 Department of Infectious Diseases, Nagasaki University Graduate School of Biomedical Sciences, 1-7-1 Sakamoto, Nagasaki 852-8501, Japan; yimamura@nagasaki-u.ac.jp (Y.I.); koizumik@nagasaki-u.ac.jp (K.I.); 3 Department of Respiratory Medicine, Nagasaki University Graduate School of Biomedical Sciences, 1-7-1, Sakamoto, Nagasaki 852-8501, Japan; 4 Clinical Research Center, Nagasaki University Hospital, 1-7-1, Sakamoto, Nagasaki 852-8501, Japan; nhosogaya@nagasaki-u.ac.jp; 5 Department of Microbiology, Toyama University Graduate School of Medicine and Pharmaceutical Sciences, Toyama 930-0194, Japan; morinaga@med.u-toyama.ac.jp; 6 Department of Laboratory Medicine, Nagasaki University Hospital, 1-7-1, Sakamoto, Nagasaki 852-8501, Japan; k-yanagi@nagasaki-u.ac.jp

**Keywords:** allergic bronchopulmonary aspergillosis, chronic pulmonary aspergillosis, serum cytokine, IL-33, IL-10/IL-5

## Abstract

Allergic bronchopulmonary aspergillosis (ABPA) and chronic pulmonary aspergillosis (CPA) are important fungal infections caused by *Aspergillus* species. An overlap of ABPA and CPA has been reported; therefore, it is critical to determine whether the main pathology is ABPA or CPA and whether antifungals are required. In this study, we investigated whether the serum cytokine profile is useful for understanding the pathology and for differentiating between these diseases. We compared the various serum cytokine levels among healthy subjects and patients diagnosed with asthma, ABPA, or CPA at Nagasaki University Hospital between January 2003 and December 2018. In total, 14 healthy subjects, 19 patients with asthma, 11 with ABPA, and 10 with CPA were enrolled. Interleukin (IL) -5 levels were significantly higher in patients with ABPA than in those with CPA, and IL-33 and tumor necrosis factor (TNF) levels were significantly higher in patients with CPA than in those with asthma (*p* < 0.05, Dunn’s multiple comparison test). The sensitivity and specificity of the IL-10/IL-5 ratio (cutoff index 2.47) for diagnosing CPA were 70% and 100%, respectively. The serum cytokine profile is useful in understanding the pathology of ABPA and CPA, and the IL-10/IL-5 ratio may be a novel supplemental biomarker for indicating the pathology of CPA.

## 1. Introduction

Allergic bronchopulmonary aspergillosis (ABPA) is a complication of asthma and cystic fibrosis, accounting for 2.5% of all asthma cases [1]. It is a disease caused by type I and type III allergies to *Aspergillus*, which causes mucus plugging, bronchiectasis, and pulmonary fibrosis mainly through eosinophilic inflammation of the airway [1,2,3,4]. Conversely, chronic pulmonary aspergillosis (CPA) is a disease caused by the saprophytic proliferation of *Aspergillus* due to structural destruction of the lungs and deterioration of host immunity [5,6]. Some of these diseases patients can be refractory to treatment and can be fatal, especially if initiation of the treatment is delayed.

In recent years, clinicians have suggested a continuity of ABPA and CPA, and an overlap of these diseases has also been reported [7,8,9,10]. Current first-line treatment of ABPA is with oral corticosteroids, while that of CPA is antifungals. Out of all patients with CPA, 14.3% develop from ABPA, and patients with ABPA with fungus balls are reported to have a poor outcome [11,12]. Therefore, it is critical to determine whether the main pathology is ABPA or CPA and whether antifungals are required. Although the clinical course of the diseases, chest computed tomography (CT) findings, and immunological tests, such as total IgE, *Aspergillus*-specific IgG, or IgE and *Aspergillus* precipitating antibody, are used to diagnose these diseases, in some cases, it may be difficult to distinguish between ABPA and CPA [7,9,13,14]. In such cases, it is difficult to determine if antifungals should be initiated, and a novel supplemental biomarker for understanding the pathology of both diseases is required.

It has been suggested that different serum cytokines, namely, interleukin (IL)-4, IL-5, and IL-13 for ABPA and IL-1β, IL-10, tumor growth factor-*β*, and soluble CD40 ligand (sCD40L) for CPA, are involved in pathology, and these serum cytokine profiles may be useful in differentiating between the two diseases [15,16,17]. In this study, we measured the levels of serum cytokines in patients with ABPA and CPA and investigated whether the serum cytokine profile is useful for understanding the disease pathology.

## 2. Materials and Methods

### 2.1. Study Design and Population

Healthy subjects were enrolled as the control group, and patients with asthma were enrolled as the disease-control group in this study. We retrospectively investigated the medical records of patients diagnosed with asthma, ABPA, or CPA at Nagasaki University Hospital between January 2003 and December 2018 and compared patient backgrounds, IgE levels, and eosinophil counts. We measured the serum cytokine levels in patients with ABPA and CPA using the stored serum samples obtained at the time of diagnosis of ABPA and CPA. In healthy volunteers (controls) and patients with asthma, serum samples were collected at the time of their entry into the study. These serum samples were dispensed into five microtubes per sample immediately after blood collection and stored at −80 °C; one microtube was used for each test to prevent repeated freeze–thaw.

Chest CT findings of patients with ABPA and CPA were evaluated by three pulmonologists with 9, 17, and 28 years of chest imaging experience, respectively. Asthma was diagnosed according to the Global Initiative for Asthma guidelines [18]. The asthma severity of patients in the disease group was classified as mild, 9; moderate, 8; or severe, 2. ABPA was diagnosed according to the International Society for Human and Animal Mycology criteria proposed in 2013 and via definite ABPA/allergic bronchopulmonary mycosis (ABPM) according to the Japan ABPM Research Program criteria proposed in 2021 [14,19]. All patients with ABPA were positive for *Aspergillus*-specific IgE antibody, and in detail, 1 patient in Class 1 (0.35–0.70 U_A_/mL), 2 in Class 2 (0.70–3.50 U_A_/mL), 4 in Class 3 (3.50–17.49 U_A_/mL), and 4 in Class 4 (17.5–50.0 U_A_/mL). The diagnosis of CPA was based on compatible clinical symptoms, radiological findings, and mycological examinations, such as positive *Aspergillus* precipitating antibody and the positive isolation of *Aspergillus* species from respiratory tract samples [5]. CPA patients included 1 with single aspergilloma and 9 with chronic cavitary pulmonary aspergillosis. *Aspergillus* precipitating antibody was negative in 2 patients, weak positive in 1 patient, and strongly positive in 7 patients. The two negative patients of *Aspergillus* precipitating antibody were cultured for non-fumigatus *Aspergillus* from bronchoalveolar lavage fluid. The diagnosis of CPA in these negative patients of *Aspergillus* precipitating antibody was performed based on strongly positive *Aspergillus* galactomannan (GM) antigen in bronchoalveolar lavage fluid. Exclusion criteria comprised being treated with systemic steroids within 6 months prior to being enrolled in this study; treatment with immunosuppressive agents; and the presence of interstitial pneumonia, lung cancer, or nontuberculous mycobacteria.

### 2.2. Measurements

*Aspergillus*-specific IgE were measured by ImmunoCAP (Thermo Fisher Scientific, Uppsala, Sweden). The Platelia *Aspergillus* enzyme immunoassay (Bio-Rad, Tokyo, Japan) was used for serum GM assays. The FSK1 *Aspergillus* immunodiffusion system (Microgen Bioproducts Ltd., Camberley, UK) was used for *Aspergillus* precipitating antibody assays in serum, according to the manufacturer’s instructions.

### 2.3. Cytokine Measurements

Serum IL-1β, IL-4, IL-6, IL-10, IL-17A, IL-17F, IL-23, IL-25, IL-33, interferon-γ (IFN-γ), tumor necrosis factor (TNF), and sCD40L levels were quantified using the Bio-Plex Pro Human Th17 Cytokine Assays kit (Bio-rad Laboratories, Hercules, CA, USA), and IL-2, IL-5, IL-8, and IL-13 levels were quantified using the Bio-Plex Pro Human Cytokine Group I Assay kit (Bio-rad Laboratories, Hercules, CA, USA) according to the manufacturer’s instructions. Cytokine measurements were performed twice.

### 2.4. Statistical Analyses

All statistical analyses were carried out using the Prism software package (version 9.0; GraphPad Software, Inc., La Jolla, CA, USA), and data are expressed as mean ± standard deviation. If the serum cytokine levels were under detectable thresholds, the threshold values were applied for analysis in those samples. The patients’ age, body mass index, IgE, eosinophil count, GM antigen, and serum cytokine levels were analyzed using the Mann–Whitney U test for comparison of two groups and with the Kruskal–Wallis test and Dunn’s multiple comparison post-test for comparison of multiple groups. The ratio of male, smoker, complications, and chest CT findings were compared using Fisher’s exact test. Statistical significance was set at *p* < 0.05. Receiver operating characteristic (ROC) curve analyses were performed on the serum cytokine ratio, and areas under the curves, including 95% confidence intervals, were evaluated to assess a supplemental biomarker for understanding the pathology of ABPA and CPA. The Youden index was used to determine the optimized cutoff in the ROC curve analysis.

### 2.5. Ethics Statement

The ethics committee of Nagasaki University Hospital approved the study protocol (#17112025-7), and all participants received verbal and written study information before providing their written informed consent.

## 3. Results

### 3.1. Characteristics of Study Participants

During the study period, 14 healthy (control) subjects, 19 patients with asthma, 11 with ABPA, and 10 with CPA were enrolled. None of the CPA patients fulfilled the ABPA diagnostic criteria of the International Society for Human Animal Mycology or the ABPM diagnostic criteria of the Japan ABPM Research Program criteria. The patients with ABPA showed significantly higher IgE levels and eosinophil counts than the other patients (*p* < 0.05) (Table 1). The patients with CPA were significantly older and had a significantly lower body mass index than those with ABPA (*p* < 0.05) (Table 1). In addition, the patients with CPA showed significantly higher GM antigen levels than those with ABPA (*p* = 0.0015). In the chest CT findings, mucoid impaction was observed in 91% (*p* < 0.0001) of patients with ABPA, and cavitary lesions and fungus balls were observed in 100% (*p* < 0.0001) and 80 % (*p* = 0.0002) of patients with CPA, respectively (Table 1).

### 3.2. Comparison of Cytokines

**Figure 1:**
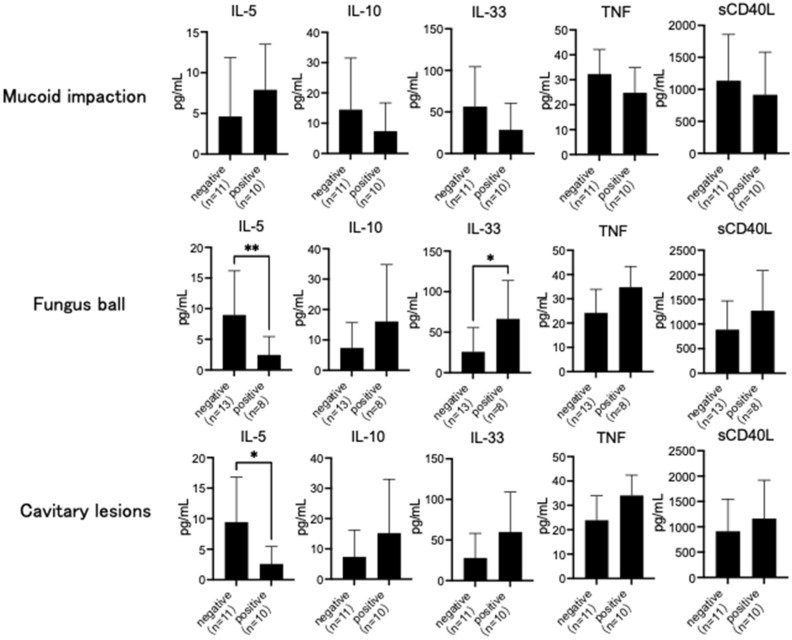
Chest computed tomography (CT) findings and serum cytokine. Interleukin (IL)-5 levels were high in subjects with mucoid impaction and significantly lower in those with fungus ball and cavitary lesions on chest CT. IL-10, IL-33, tumor necrosis factor (TNF), and soluble CD40 ligand (sCD40L) levels were low in subjects with mucoid impaction and high in those with cavitary lesions on chest CT. IL-33 level was significantly higher in subjects with fungus balls. Data are expressed as the mean ± standard deviation. Error bars indicate standard deviation. Statistical analyses were performed using the Mann–Whitney U test. Asterisks indicate statistically significant differences (* *p* < 0.05; ** *p* < 0.01).

IL-1β, IL-4, IL-17A, and IL-17F levels were under the detection threshold in all subjects. While IFN-γ was detected only in patients with CPA, IL-6 and IL-8 levels were the highest in those with ABPA. On the other hand, IL-10, IL-23, IL-25, and sCD40L levels were the highest in patients with CPA. IL-5 levels were significantly higher in patients with asthma and ABPA than in those with CPA (*p* < 0.05) (Table 2), and IL-13 levels were significantly higher in patients with asthma than in those with CPA (*p* < 0.05) (Table 2). IL-33 and TNF levels were significantly higher in patients with CPA than in those with asthma (*p* < 0.05) (Table 2). However, IL-5 levels were high in patients with mucoid impaction and significantly lower in patients with fungus balls and cavitary lesions on chest CT (*p* < 0.05) (Figure 1). IL-10, IL-33, TNF, and sCD40L levels were low in patients with mucoid impaction and high in those with cavitary lesions on chest CT. IL-33 level was significantly higher in patients with fungus balls (*p* < 0.05) (Figure 1).

### 3.3. Cytokine Ratios

**Figure 2:**
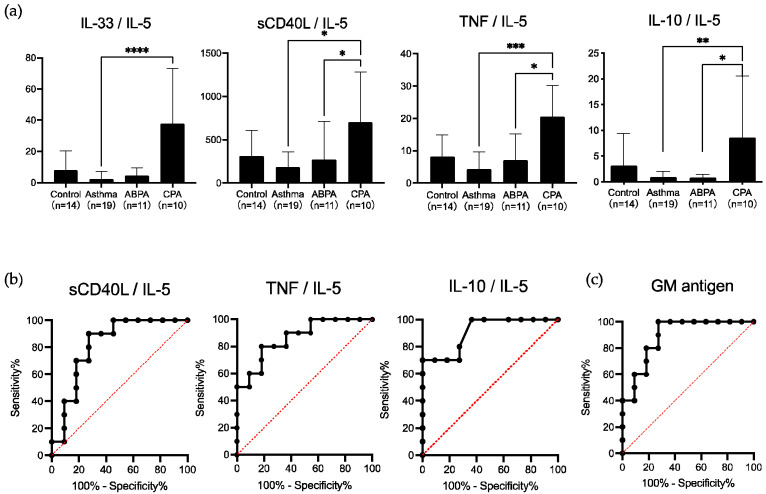
Serum cytokine ratio and receiver operating characteristic (ROC) curves. (**a**) The Interleukin (IL)-33/IL-5 ratio was not significantly different between allergic bronchopulmonary aspergillosis (ABPA) and chronic pulmonary aspergillosis (CPA) subjects, but the soluble CD40 ligand (sCD40L)/IL-5, tumor necrosis factor (TNF)/IL-5, and IL-10/IL-5 ratios were significantly higher in subjects with CPA than in those with ABPA. Data are expressed as the mean ± standard deviation. Error bars indicate standard deviation. Statistical analyses were performed using the Kruskal–Wallis test with Dunn’s multiple comparison post-test. Asterisks indicate statistically significant differences (* *p* < 0.05; ** *p* < 0.01; *** *p* < 0.001; **** *p* < 0.0001). (**b**) ROC curve analyses were performed on the serum cytokine ratio, and areas under the curves, including 95% confidence intervals, were evaluated. Areas under the curves (95% confidence interval) for sCD40L/IL-5, TNF/IL-5, and IL-10/IL-5 ratios were 0.818 (0.627–1.0000), 0.864 (0.709–1.000), and 0.909 (0.786–1.000), respectively. (**c**) ROC curve analyses were performed on the galactomannan (GM) antigen, and areas under the curves, including 95% confidence intervals, were evaluated. The area under the curve (95% confidence interval) for the GM antigen was 0.891 (0.751–1.000).

To assess differences in the pathology between ABPA and CPA, we evaluated the serum cytokine ratio using IL-5, which was elevated in patients with ABPA, and IL-33 and TNF, which were elevated in those with CPA. Although IL-6 and IL-8 levels were the highest in patients with ABPA and IL-23 levels were the highest in patients with CPA, these cytokines were excluded due to the large dispersion of data within the group. Both IL-10 and sCD40L were evaluated in patients with CPA, although there were no significant differences with the other groups. As these cytokines might be related to *Aspergillus* infection, we added them to the analysis of ROC curve [15,20]. The IL-33/IL-5 ratio was not significantly different between patients with ABPA and those with CPA; however, the sCD40L/IL-5, TNF/IL-5, and IL-10/IL-5 ratios were significantly higher in patients with CPA than in those with ABPA (*p* < 0.05) (Figure 2a).

The sensitivity and specificity of the sCD40L/IL-5 ratio for diagnosing CPA were 90% and 72.7%, respectively, at a cutoff index of 176.9, optimized from ROC curves, and those of the TNF/IL-5 ratio were 80% and 81.8%, respectively, at a cutoff index of 14.68, optimized from ROC curves. The IL-10/IL-5 ratio showed lower sensitivity and higher specificity than the other two ratios, 70% and 100%, respectively, at a cutoff index of 2.47, optimized from the ROC curve (Figure 2b). The areas under the curves of the ROC curves for these three ratios were 0.818, 0.864, and 0.909, respectively (Figure 2b).

### 3.4. Usefulness of Cytokine Ratios

To evaluate whether cytokine ratios are useful as a supplemental biomarker for elucidating the pathology of ABPA and CPA, we compared these ratios with the sensitivity and specificity of various conventional markers. The total IgE, eosinophilic count, and mucoid impaction in chest CT, which are included in the ABPA diagnostic criteria for enrolling the patients with ABPA and the presence of cavitary lesions and fungus balls on chest CT, which are included in the CPA diagnostic criteria for enrolling the patients with CPA in this study, showed high sensitivity and specificity (Table 3). The GM antigen (cutoff index 0.267) had higher sensitivity and lower specificity than the IL-10/IL-5 ratio, 70% and 100%, respectively (Table 3). The area under the curves of the ROC curves of the GM antigen was 0.891, which was lower than that of the IL-10/IL-5 (Figure 2c).

## 4. Discussion

To the best of our knowledge, this is the first study to demonstrate an association between IL-33 and *Aspergillus* infection using clinical samples and to propose that serum cytokines may be useful in understanding the pathology in ABPA and CPA. In this study, we demonstrated that IL-5 levels were high in ABPA; those of IL-10, IL-33, TNF, and sCD40L were high in CPA; and the IL-10/IL-5 ratio could be a novel supplemental biomarker indicating the pathology of CPA.

Previous studies have shown that ABPA is caused by an excessive immune response to *Aspergillus* due to the release of IL-4, IL-5, and IL-13 by the Th2 CD4+ T-cell response [2,16]. These cytokines secreted from activated Th2 CD4+ T-cells lead to eosinophil activation as well as B cell differentiation. Exposure to *A. fumigatus* induces the degranulation of mast cells and basophils via IgE antibodies. Along with them, eosinophils interact directly with *A. fumigatus* conidia, produce extracellular traps, and injure the bronchial epithelium [4,21,22]. In this study, although IL-4 levels were under the detection threshold and IL-13 levels were low in patients with ABPA, their IL-5 levels were significantly higher than those in patients with CPA and were the second-highest after those in patients with asthma. These results suggest that ABPA is mediated by the Th2 response. On the other hand, the TNF levels were significantly higher in patients with CPA than in those with asthma. TNF is an important proinflammatory cytokine, as it plays a key role in the host defense during the earliest stages of *Aspergillus* infections [23,24]. High levels of TNF have been demonstrated in the lungs of mice infected intranasally or intratracheally with *A. fumigatus* conidia [25,26,27]. Although we evaluated serum cytokines, TNF levels were found to be significantly higher in patients with CPA with fungus ball on chest CT, which increase on exposure to *Aspergillus* spp. The sCD40L and IL-10 levels were higher in patients with ABPA and CPA patients. CD40L is involved in allergic responses by inducing IL-4 production from T cells and inducing IgE production from B cells [28]. The hyphae of *A. fumigatus* has been demonstrated to secrete CD40L from platelets more strongly than conidia [20]. On the other hand, CD40/CD40L is involved in local immunosuppression and immune tolerance by generating IL-10 secreting B cells [29]. Although CD40L secreted by exposure to *Aspergillus* spp causes an allergic reaction, it may eventually induce IL-10 secretion to suppress excessive immunity.

In recent years, an association between the IL-33 and *Aspergillus* infection has been reported [30,31]. IL-33 is known to be constitutively present in the nuclei of epithelial cells and is released upon exposure to viral and fungal antigens, predominantly activating type 2 innate lymphoid cells (ILC2s) and producing IL-5 and IL-13 [21,30]. This activation enhances Th2 response and reduces fungal clearance. Our study showed that IL-33 levels were elevated in patients with ABPA and CPA and that IL-33 levels were notably higher in patients with CPA than in those with asthma and significantly higher in those with CPA with fungus balls on chest CT. Considering that the GM antigen was significantly elevated in patients with CPA, these results suggest that IL-33 is secreted by exposure to *Aspergillus* spp.

Additionally, in patients with ABPA, IL-5 levels were also found to be elevated, suggesting a shift to Th2 balance; however, in patients with CPA, both IL-5 and IL-13 levels were low, despite the high level of IL-33. This response in patients with CPA may be affected by IL-10, which is a major immunosuppressive cytokine that functions as a homeostatic host-driven response to control inflammation, which reduces damage to the host. IL-33 has been reported to induce IL-10 producing immunosuppressive ILC2 with retinoic acid, and severe allergic inflammation is known to induce exhausted-like ILC2s with high expression of IL-10 and low expression of IL-5 and IL-13 [32,33]. In cases where CPA develops from ABPA, ILC2 exhaustion caused by an excessive allergic reaction may allow *Aspergillus* to rot into the lungs. Given the movement of these cytokines, the IL-10/IL-5 ratio may be a supplemental biomarker indicating the pathology of CPA.

This study had some limitations. First, the number of enrolled patients was small. Second, some of the patients had underlying diseases which might be attributed to the serum cytokine profiles. Third, all of the characteristics of the study participants, such as age, gender, body mass index, and smoking history may affect the specific serum cytokine profiles, and may confound the finding of differences in certain cytokines between groups. Fourth, since the collection time of each patient’s serum is different, serum cytokine levels may be affected by the stock duration of serum samples [34]. However, it is a common finding that patients with ABPA and CPA have some sort of underlying disease, and the results of this study can be applied in clinical practice.

## 5. Conclusions

This is the first report to demonstrate the association between IL-33 and *Aspergillus* infection using clinical samples and the usefulness of the serum cytokine profile in understanding the pathology in ABPA and CPA. Our results might help in distinguishing between ABPA and CPA and in determining when to initiate antifungals. A large-scale prospective study to investigate cytokine changes in patients with ABPA or CPA is required to further elucidate our findings. Further studies on the association between *Aspergillus* infection and immunity may lead to the use of anti-cytokine antibodies in the treatment of ABPA and CPA in the future.

## Figures and Tables

**Table 1:** Characteristics of Study Participants.

	Control (*n* = 14)	Asthma (*n* = 19)	ABPA (*n* = 11)	CPA (*n* = 10)	*p*-Value
Age (years)	37 ± 7.626	56.7 ± 15.9 *	47.1 ± 13.3	66.5 ± 10.6 *^,^***	***p* < 0.0001**
Male, *n* (%)	5 (35.7)	5 (26.3)	4 (36.3)	8 (80.0)	***p* = 0.0410**
Body mass index (kg/m^2^)	20.8 ± 3.26	23.8 ± 4.63	21.4 ± 3.42	18.0 ± 2.67 **^,^***	***p =* 0.0007**
Smoker, *n* (%)	6 (42.8)	3 (15.7)	5 (45.4)	8 (80)	***p* = 0.0097**
IgE (IU/mL)	122.0 ± 263.2	491.2 ± 944.0	3694 ± 3451 ****	105.1 ± 184.8	***p* < 0.0001**
Eosinophil count (/μL)	-	275.2 ± 238.4	1198 ± 597.5 ****	158.5 ± 103.4	***p* < 0.0001**
Galactomannnan (C.O.I)	-	-	0.33 ± 0.43	2.22 ± 3.21	***p* = 0.0015**
*Aspergillus* precipitating antibody, *n* (%)	-	-	4 (36.3)	8 (80.0)	*p* = 0.0805
Complications, *n* (%)					
COPD	-	2 (10.5)	0	3 (30)	*p* = 0.1870
Bronchiectasis	-	0	7 (63.6)	4 (40)	***p* = 0.0005**
Tuberculosis sequelae	-	0	0	1 (10)	*p =* 0.4762
Post-thoracic surgery	-	0	1 (9)	2 (20)	*p =* 0.5865
Lung cyst	-	0	0	1 (10)	*p =* 0.4762
Diabetes mellitus	-	2 (10.5)	2 (18.2)	0	*p* = 0.6111
Chest CT findings					
mucoid impaction, *n* (%)	-	-	10 (91)	0	***p <* 0.0001**
Consolidation, *n* (%)	-	-	7 (63.6)	5 (50)	***p* = 0.0003**
Bronchiectasis, *n* (%)	-	-	7 (63.6)	5 (50)	***p =* 0.0003**
Cavitary lesions, *n* (%)	-	-	0	10 (100)	***p <* 0.0001**
Fungus balls, *n* (%)	-	-	0	8 (80)	***p =* 0.0002**

ABPA, allergic pulmonary aspergillosis; CPA, chronic pulmonary aspergillosis; COPD, chronic obstructive pulmonary disease; CT, computed tomography. Data are expressed as the mean ± standard deviation. A *p*-value < 0.05 (Kruskal–Wallis test, Fisher’s exact test, or Mann–Whitney U test) was considered significant. Values in boldface are significant. Asterisks indicate statistical significance using the Dunn’s multiple comparison post-test (* *p* < 0.05 vs. control; ** *p* < 0.05 vs. asthma; *** *p* < 0.05 vs. ABPA; **** *p* < 0.05 vs. the others).

**Table 2:** Serum cytokine levels.

	Control (*n* = 14)	Asthma (*n* = 19)	ABPA (*n* = 11)	CPA (*n* = 10)	*p*-Value
IL-1β	N.D.	N.D.	N.D.	N.D.	-
IL-2	N.D.	1.80 ± 0.73	N.D.	1.73 ± 0.54	*p* = 0.4413
IL-4	N.D.	N.D.	N.D.	N.D.	-
IL-5	4.08 ± 2.92	11.46 ± 16.65 ***	9.44 ± 7.37 ***	2.60 ± 2.84	***p* = 0.0099**
IL-6	N.D.	0.81 ± 0.53	90.35 ± 295.9	6.26 ± 4.23 **	***p* < 0.0001**
IL-8	16.36 ± 7.40	42.01 ± 37.61	709.2 ± 1479 *	61.25 ± 53.11 *	***p* = 0.0065**
IL-10	10.85 ± 24.13	3.12 ± 2.02	7.35 ± 8.86	15.23 ± 17.75	*p* = 0.2573
IL-13	0.40 ± 0.19	2.13 ± 2.37 *^,^***	0.63 ± 0.56	0.42 ± 0.22	***p* = 0.0032**
IL-17A	N.D.	N.D.	N.D.	N.D.	-
IL-17F	N.D.	N.D.	N.D.	N.D.	-
IL-23	29.69 ± 13.18	N.D.	50.49 ± 46.47	155.9 ± 358.5	*p* = 0.1022
IL-25	0.51 ± 0.28	0.46 ± 0.51	0.78 ± 0.69	1.16 ± 1.00	***p* = 0.0348**
IL-33	18.19 ± 19.16	6.07 ± 9.73	27.97 ± 30.23	59.91 ± 49.12 **	***p* = 0.0002**
IFN-γ	N.D.	N.D.	N.D.	5.26 ± 6.05	***p* = 0.0297**
TNF	19.19 ± 8.27	20.42 ± 11.7	23.85 ± 10.19	34.05 ± 8.36 *^,^**	***p* = 0.0021**
sCD40L	735.6 ± 368.5	694.1 ± 202.7	912.4 ± 632.5	1162 ± 757.1	*p* = 0.2740

ABPA, allergic pulmonary aspergillosis; CPA, chronic pulmonary aspergillosis; IL, interleukin; IFN, interferon; TNF, tumor necrosis factor; sCD40L, soluble CD40 ligand. Data are expressed as the mean ± standard deviation. A *p*-value < 0.05 (Kruskal–Wallis test or Mann–Whitney U test) was considered significant. Values in boldface are significant. Asterisks indicate statistical significance using the Dunn’s multiple comparison post-test (* *p* < 0.05 vs. control; ** *p* < 0.05 vs. asthma; *** *p* < 0.05 vs. CPA).

**Table 3:** Diagnostic performance of markers for ABPA and CPA.

Diagnostic Markers	Value (%)			
	Sensitivity	Specificity	Positive Predictive Value	Negative Predictive Value
ABPA				
IgE (cutoff, 602.1 IU/mL)	100	100	100	100
Eosinophil count (cutoff, 441.0/μL)	100	100	100	100
Chest CT findings				
mucoid impaction	100	100	100	100
CPA				
Age (cutoff, 61 years)	70	81.8	77.8	75
Body mass index (cutoff, 19.41 kg/m^2^)	90	72.7	75.0	88.9
Galactomannnan (cutoff index, 0.267)	90	72.7	75.0	88.9
Chest CT findings				
Cavitary lesions	100	100	100	100
Fungus balls	80	100	100	84.6
Cytokine ratios				
sCD40L/IL-5 (cutoff, 176.9)	90	72.7	75.0	88.9
TNF/IL-5 (cutoff, 14.68)	80	81.8	81.8	75
IL-10/IL-5 (cutoff, 2.47)	70	100	100	78.6

ABPA, allergic pulmonary aspergillosis; CPA, chronic pulmonary aspergillosis; CT, computed tomography; sCD40L, soluble CD40 ligand; IL, interleukin; TNF, tumor necrosis factor.

## Data Availability

Not applicable.
